# Transient gap generation in BaFe_2_As_2_ driven by coherent lattice vibrations

**DOI:** 10.1093/pnasnexus/pgad164

**Published:** 2023-05-16

**Authors:** Jacob A Warshauer, Daniel Alejandro Bustamante Lopez, Qingxin Dong, Genfu Chen, Wanzheng Hu

**Affiliations:** Department of Physics, Boston University, 590 Commonwealth Avenue, Boston, 02215 MA, USA; Department of Physics, Boston University, 590 Commonwealth Avenue, Boston, 02215 MA, USA; Institute of Physics and Beijing National Laboratory for Condensed Matter Physics, Chinese Academy of Sciences, 8 Zhongguancun 3rd South Street, 100190 Beijing, China; School of Physical Sciences, University of Chinese Academy of Sciences, No. 19 A Yuquan Road, 100049 Beijing, China; Institute of Physics and Beijing National Laboratory for Condensed Matter Physics, Chinese Academy of Sciences, 8 Zhongguancun 3rd South Street, 100190 Beijing, China; School of Physical Sciences, University of Chinese Academy of Sciences, No. 19 A Yuquan Road, 100049 Beijing, China; Department of Physics, Boston University, 590 Commonwealth Avenue, Boston, 02215 MA, USA; Division of Materials Science and Engineering, Boston University, 590 Commonwealth Avenue, Boston, 02215 MA, USA

**Keywords:** iron-based superconductors, optical properties, nonlinear dynamics

## Abstract

Iron-based superconductors provide a rich platform to investigate the interplay between unconventional superconductivity, nematicity, and magnetism. The electronic structure and the magnetic properties of iron-based superconductors are highly sensitive to the pnictogen height. Coherent excitation of the $A_{1g}$ phonon by femtosecond laser directly modulates the pnictogen height, which has been used to control the physical properties of iron-based superconductors. Previous studies show that the driven $A_{1g}$ phonon resulted in a transient increase of the pnictogen height in BaFe${}_{2}$As${}_{2}$, favoring an enhanced Fe magnetic moment. However, there are no direct observations on either the enhanced Fe magnetic moments or the enhanced spin-density wave (SDW) gap. Here, we use time-resolved broadband terahertz spectroscopy to investigate the dynamics of BaFe${}_{2}$As${}_{2}$ in the $A_{1g}$ phonon-driven state. Below the SDW transition temperature, we observe a transient gap generation at early-time delays. A similar transient feature is observed in the normal state up to room temperature.

**Significance Statement**

Our work makes a new fundamental experimental discovery on a transient gap induced in the parent compound of iron-based superconductors by modulating the iron–arsenic distance with laser excitations. The transient gap is a large effect, which develops in the spin-density wave state and persists up to room temperature. The transient gap appears in the early stages of the photoexcited state with a shorter lifetime in comparison with the other transient feature. Our finding is the missing patchwork to complete the nonequilibrium picture of iron pnictides in the phonon-driven state, opening up new possibilities to study the impact of lattice distortions on coexisting orders in unconventional superconductors.

Controlling the physical properties of quantum materials along noninvasive and ultrafast pathways is the key for developing next-generation technologies. Dynamical control of materials using ultrashort laser pulses has been successful in a variety of systems to achieve novel phases that are inaccessible at equilibrium (1–4). The key is to identify tuning parameters which effectively modify the electronic structure of quantum materials.

Quantum materials are remarkably sensitive to structural distortion. In iron pnictides, the iron–arsenic distance (pnictogen height) has a significant impact on superconductivity (5, 6), the electronic band structure (7–10), and the magnetic properties (11, 12). The pnictogen height can be periodically modulated by optical excitation of a Raman-active $A_{1g}$ phonon (Fig. 1A) (13, 14). For BaFe${}_{2}$As${}_{2}$, a displacive excitation towards larger pnictogen height is observed in the transient state (15, 16, 9), which favors an enhanced Fe magnetic moment. One would expect to see a displacive increase of the spin-density wave (SDW) gap size, but this has not been observed so far due to experimental challenges. In fact, a comprehensive band assignment and gap identification at equilibrium are already challenging (17–20) due to the multiband nature of iron pnictides: the energy bands close to the Fermi energy come from three orbitals, which experience strong band hybridization and band splitting below the nematic ordering temperature. This situation is further complicated by the twinned domains in as-grown samples (21, 22). In the transient state, the $A_{1g}$ phonon is excited by femtosecond laser pulses in the near-infrared, the energy scale of which is far above that of the SDW gap. This results in a large contribution from the photoexcited carriers in the phonon-driven state, which may wash out low-energy features such as the SDW gap.

**Fig. 1.**
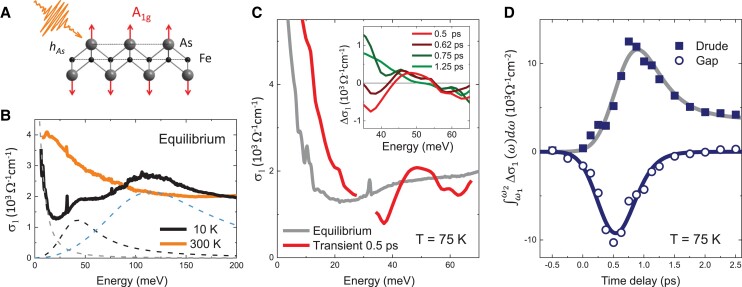
Equilibrium and transient optical response for BaFe${}_{2}$As${}_{2}$ in the SDW state. A) Coherent excitation of the $A_{1g}$ phonon modulates the pnictogen height $h_{As}$. B) Optical evidence of SDW gap formation at equilibrium: $\sigma_{1}(\omega )$ at T=10 K shows peaks at 45 and 110 meV, representing the SDW gaps (26). Solid lines are $\sigma_{1}(\omega )$ at temperatures below and above $T_{SDW}$. Dashed lines are the Drude term and two Lorentz peaks from a fit for *T* = 10 K. C) (Main) Transient optical conductivity at T=75 K. The transient $\sigma_{1}(\omega )$ shows a broadened Drude component and a gap formation near 50 meV which takes the spectral weight from the equilibrium SDW gap at 45 meV. The data discontinuity at 30 meV is due to a detection gap in the pump-probe setup. The pump fluence (incident fluence) is 0.53 mJ/cm${}^{2}$. (Inset) Light-induced change in the real part of optical conductivity, $\Delta \sigma_{1}(\omega )=\sigma_{1}(\omega {)}^{\mathrm{transient}}-\sigma_{1}(\omega {)}^{\mathrm{equilibrium}}$, at early-time delays. The transient gap reaches its maximum earlier than the transient Drude component. D) Different time scales for the light-induced Drude component (solid squares) and the transient gap (open circles) are further evidenced by the time evolution of the transient change in spectral weight, $\int_{\omega_{1}}^{\omega_{2}}\Delta \sigma_{1}(\omega )\;d\omega$, where $\omega_{1}=34$ meV and $\omega_{2}=43$ meV. Solid lines are exponential fits.

A remarkable result is from a recent time-resolved optical spectroscopy study, reporting a transient feature resembling the SDW gap at a temperature slightly above the SDW transition temperature $T_{SDW}$ (23). This was attributed to the generation of SDW order due to modified iron–arsenic distance in the phonon-driven state. However, it is unclear why the transient SDW order, which is so robust that exists up to room temperature, doesn’t exist in the SDW state when driving the same $A_{1g}$ phonon, as the lattice and band structure modifications are along the same direction for temperatures above and below $T_{SDW}$ (15, 16, 9). Furthermore, as the transient gap was obtained from the averaged oscillation amplitude of the optical conductivity, it remains unknown whether a corresponding behavior exists in the displacive response to counter the enhanced pnictogen height in the phonon-driven state (15, 16).

Here, we report a time-resolved broadband terahertz (THz) spectroscopic probe of BaFe${}_{2}$As${}_{2}$ in the $A_{1g}$ phonon-driven state. The THz probe covers a spectral range from 8 to 70 meV, which allows the detection of light-induced changes in the itinerant carriers and the low-energy SDW gap. We studied the time and temperature dependence of the transient optical conductivity $\sigma_{1}(\omega )$. Below $T_{SDW}$, we observed a light-induced depletion of the optical conductivity at the equilibrium SDW gap, with a peak forming at higher energies. This is a clear optical signature of gap opening. This feature is observed at early pump-probe time delays, when the photoexcited carriers are accumulating. The transient gap is quickly filled and merges to a free carrier response at later delays. Temperature-dependent pump-probe measurements show that a similar transient gap develops above $T_{SDW}$ and persists up to room temperature. The direct observation of a transient gap from the displacive response at temperatures both below and above $T_{SDW}$ provides new insights into the physics of iron pnictides, and will stimulate promising experiments in the optically induced control of nonequilibrium states of matter.

## Results

The BaFe${}_{2}$As${}_{2}$ single crystals exhibiting an SDW transition at $T_{SDW}=130$ K were grown by self-flux method (see Supplementary Fig. S1) (24). Near-infrared (800 nm) laser pulses with a 40 fs duration were used to excite the $A_{1g}$ phonon in BaFe${}_{2}$As${}_{2}$. The transient optical properties were probed at normal incidence by broadband THz pulses generated by laser-ionized plasma (25). The THz pulses were detected by electrooptical (EO) sampling of the terahertz field in a 100 micron thick GaP and a 300 micron thick GaSe crystal, which cover a detection range from 8 to 28 meV and from 34 to 70 meV, respectively.

The equilibrium optical conductivity $\sigma_{1}(\omega )$, above and below $T_{SDW}$, is shown in Fig. 1B. In the SDW state, several features are seen in $\sigma_{1}(\omega )$: a Drude term at low frequencies, representing the free-carrier response; a sharp peak at 32 meV from an $E_{u}$ infrared-active phonon (26, 27); and two peaks at 45 and 110 meV, representing the SDW gaps (26, 18, 20).

Fig. 1C shows the transient $\sigma_{1}(\omega )$ at T=75 K. At a time delay of 0.5 ps, $\sigma_{1}(\omega )$ shows a broadening of the low-frequency Drude component with an enhanced spectral weight. This indicates a higher carrier scattering rate and an increased carrier density, which are from photoexcited carriers since BaFe${}_{2}$As${}_{2}$ is a metal. In the high-frequency region, the transient $\sigma_{1}(\omega )$ is suppressed at around 40 meV, and then goes above the equilibrium $\sigma_{1}(\omega )$ and peaks at 50 meV. This is an optical fingerprint of gap opening.

In the following, we will focus on the high-frequency region which probes both the transient gap and the tail of the Drude component. The inset of Fig. 1C plots the light-induced change in the optical conductivity at selected time delays, which shows that the Drude broadening and the transient gap evolve with different time constants. The transient gap formation is maximized at 0.5 ps, and then weakens with increasing time delay. Meanwhile, a transient Drude contribution develops, which brings $\Delta \sigma_{1}(\omega )$ to positive values near 40 meV after 0.62 ps, and reaches maximum after 0.75 ps. At 1.25 ps, the transient gap disappears, and $\Delta \sigma_{1}(\omega )$ contains only a Drude term, which decays with time. Change in optical conductivity over the entire frequency range across extended delays is seen in Supplementary Fig. S2.

To extract the time evolution of the transient gap and the Drude component, we integrated the light-induced change in the real part of optical conductivity $\int_{\omega_{1}}^{\omega_{2}}\Delta \sigma_{1}(\omega )\;d\omega$ over the frequency region in which the transient depletion of $\sigma_{1}(\omega )$ occurs: $\omega_{1}=34$ meV to $\omega_{2}=43$ meV. By fitting the transient spectral weight (see Supplementary material), we separated the contributions of the transient gap and the transient Drude component. In Fig. 1D, the open circles represent the transient gap, which causes a suppression in $\int_{\omega_{1}}^{\omega_{2}}\Delta \sigma_{1}(\omega )\;d\omega$, and the solid squares represent the transient Drude response, which lifts up $\int_{\omega_{1}}^{\omega_{2}}\Delta \sigma_{1}(\omega )\;d\omega$ to positive values in the same frequency region. It is clear that the transient gap and the Drude component develop with different time constants. The spectral weight suppression at 40 meV reaches maximum at 0.5 ps, when the Drude component from photoexcited carriers is still on the rise. At around 0.8 ps, the transient gap is significantly weakened, and the Drude component reaches maximum. The transient gap decays with a shorter lifetime (0.19 ps) than that of the Drude component (0.44 ps). The existence of two time scales is consistent with previous pump-probe studies (7, 28). Different time scales of the transient gap and Drude component suggest that they are separated nonequilibrium processes from different bands.

**Fig. 2.**
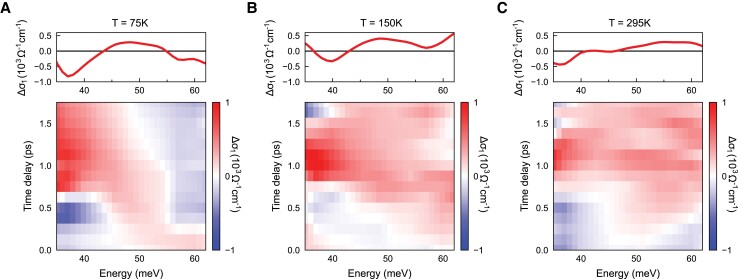
Temperature evolution of the transient gap with a pump fluence of 0.53 mJ/cm${}^{2}$. A) $\Delta \sigma_{1}(\omega )$ in the SDW state at T=75 K. B) $\Delta \sigma_{1}(\omega )$ in the normal state at T=150 K. C) $\Delta \sigma_{1}(\omega )$ at room temperature. The color plots are $\Delta \sigma_{1}(\omega )$ at early pump-probe delays; the top panels show $\Delta \sigma_{1}(\omega )$ at t=0.5 ps. The transient gap generation is seen from below to above the SDW transition temperature.

We now focus on the early-time optical response to investigate the temperature dependence of the transient gap. Fig. 2 presents $\Delta \sigma_{1}(\omega )$ from 0 to 1.8 ps at three temperatures. The transient gap formation is seen from below to above $T_{SDW}$ and persists up to room temperature. The transient gap develops at nearly the same energy for all temperatures, with weakened features at higher temperatures. The lifetime of the transient gap remains the same for below and above $T_{SDW}$ (see Supplementary Table S1 and Fig. S4).

**Fig. 3.**
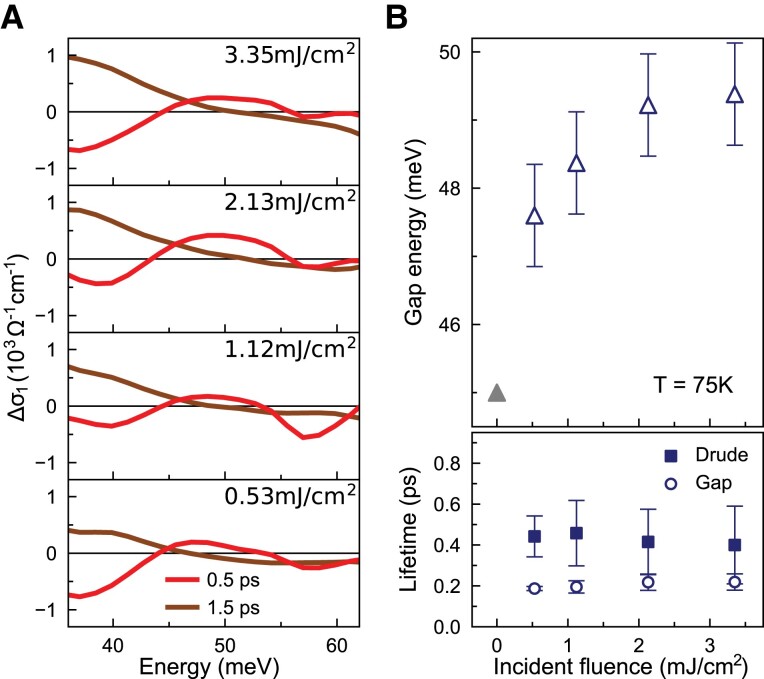
Pump fluence dependence of the transient state at T=75 K. A) $\Delta \sigma_{1}(\omega )$ at two characteristic delays for various pump fluences. B) With increasing pump fluences, the transient gap shifts to higher energies (upper panel), while the time constants of the transient state remain nearly the same (lower panel).

The pump fluence dependence of the transient state is shown in Fig. 3A. Using the peak position in $\sigma_{1}(\omega )$ to define the gap energy, we obtained the small SDW gap size as 45 meV (Fig. 1B). Similarly, we identified the transient gap size from Fig. 3A using the peak position in $\Delta \sigma_{1}(\omega )$. At 0.5 ps time delay, a transient gap develops near 48 meV with a 0.53 mJ/cm${}^{2}$ pump fluence. The gap moves to higher energies with increasing fluences. When the pump fluence is approaching 3 mJ/cm${}^{2}$, the gap energy saturates (Fig. 3B upper panel). With the same procedure used for Fig. 1D, we analyzed the time evolution of $\Delta \sigma_{1}(\omega )$ for each pump fluence (see Supplementary Fig. S3). The extracted time constants are shown in the lower panel of Fig. 3B. The lifetimes of the transient gap and the Drude component remain nearly unchanged with increasing pump fluences.

**Fig. 4.**
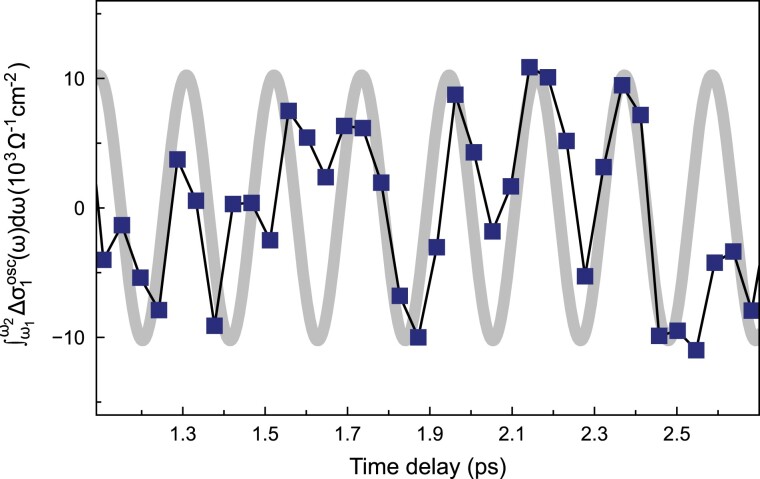
The phonon-driven state evidenced by the oscillatory response in the optical conductivity. The squares are the integrated $\int_{\omega_{1}}^{\omega_{2}}\Delta \sigma_{1}(\omega )\;d\omega$, with the displacive component subtracted. Here, $\omega_{1}=12$ meV and $\omega_{2}=20$ meV. The data were taken at T=120 K with a pump fluence of 0.53 mJ/cm${}^{2}$. A sinusoidal function with a 5-THz oscillation frequency is shown as a guide to the eye.

So far we have demonstrated a displacive response of optical conductivity in the phonon-driven state. We now present the oscillatory response. Since the electronic band structure oscillates at the frequency of the driven phonon (9, 29), the same oscillation should show up in the spectral weight of optical conductivity, as $\omega \sigma_{1}(\omega )$ is proportional to the joint density of states (30). However, it is challenging to spectrally resolve the oscillatory response: it is a weak modulation on top of the large background displacive response. In addition, the signal-to-noise and time resolution in the terahertz region are orders of magnitude worse than that in the near-infrared region (23). To minimize the effect from the strong displacive response, fine time delay scans with a 2.5 ps time window were carried out for the low-frequency region, where the signal-to-noise allows a quantitative analysis of the oscillation component. Fig. 4 shows the time evolution of the integrated transient conductivity change, $\int_{\omega_{1}}^{\omega_{2}}\Delta \sigma_{1}(\omega )\;d\omega$, with the displacive response subtracted. The spectral weight integral was evaluated with $\omega_{1}=12$ meV and $\omega_{2}=20$ meV. The gray line is an sinusoidal oscillation at 5 THz, serving as a guide to the eye. The oscillation is better resolved at later delays, when the transient gap disappears. Considering the limited time resolution of the low-frequency THz probe (160 fs), the oscillation frequency agrees qualitatively with the $A_{1g}$ phonon frequency probed by Raman spectroscopy (31, 18), and is consistent with the oscillation observed by previous pump-probe studies (32, 23, 8, 9, 16, 29, 33). Similar oscillation is also observed in the high-frequency region where the transient gap develops (see Supplementary Fig. S5). The oscillatory response of optical conductivity verifies that the transient state we studied is a $A_{1g}$ phonon-driven state.

## Discussion

We now discuss the possible origin of the light-induced gap at 50 meV. A time-resolved photoemission study of BaFe${}_{2}$As${}_{2}$ reported a displacive downwards shifting of the chemical potential by 50 meV at temperatures both below and above $T_{SDW}$ (9). However, a chemical potential shift will lead to a gap feature only when it creates additional interband transitions. Note that the pump fluence used here is the incident fluence, which is a factor of 2 larger than the corresponding absorbed fluence. Since the chemical potential shift scales linearly with the pump fluence (9), we estimated a 20-meV chemical potential shift with a 0.53 mJ/cm${}^{2}$ incident fluence. From the band structure of BaFe${}_{2}$As${}_{2}$ (22), a 20-meV chemical potential shift is insufficient to create additional interband transitions.

The transient gap is likely from a displacive band reconstruction as a consequence of a transiently increased pnictogen height. Time-resolved X-ray diffraction studies observed a displacive increase of the Fe–As distance in the phonon-driven state. The maximum transient increase of the pnictogen height, considering the sum of the displacive and oscillatory components, is more than 5% of the equilibrium pnictogen height with an absorbed pump fluence of 3.5 mJ/cm${}^{2}$ (16, 15). As the electronic structure and the magnetic properties of iron pnictides are highly sensitive to the pnictogen height (16, 11, 12), a significant structural modification would visibly affect the SDW order. The lifetimes of both the displaced pnictogen height (15) and the driven $A_{1g}$ phonon (9) show no systematic variation with pump fluences, which agrees with the fluence independent lifetime of the transient gap. Below $T_{SDW}$, since the transient gap takes the spectral weight from the equilibrium SDW gap (Fig. 1C), they possibly share the same origin: the transient gap is a blue-shifted SDW gap. This is consistent with the previous observations that an increased pnictogen height favors an enhanced Fe magnetic moment and an enhanced SDW transition temperature (16, 15).

Kim et al. (23) report a high-energy SDW gap-like feature obtained from the oscillatory response of the optical conductivity at temperatures above $T_{SDW}$. In fact, with a displacive increase of the Fe–As distance in the phonon-driven state, a light-enhanced SDW is expected in both the oscillatory and displacive responses. In addition, a transient SDW gap robust enough to survive from $T_{SDW}$ up to room temperature is expected to persist for $T<T_{SDW}$, since the lattice displacement and band modulation induced by the driven phonon were observed for both below and above $T_{SDW}$ (16, 15, 9). Our result is the first time-resolved optical study focusing on the transient response of the low-frequency SDW gap, which shows persistent gap opening from the displacive response of optical conductivity, and for temperatures both below and above $T_{SDW}$.

Note that the transient gap develops and decays with different time scales than that of the transient Drude component (Fig. 1C inset and Fig. 1D). This can be understood as the following: the transient Drude comes from the thermalization of photoexcited carriers, which is commonly seen for metals (34). It is a separated process from the transient modification of the lattice structure and band structure (9, 15) which lead to the transient gap. Since iron pnictides are multiband materials, the coexistence of photoexcited carriers challenges the detection of the transient gap. Here, time-resolved optical spectroscopy has advantages in resolving the transient gap from the background of photoexcited carriers thanks to its high-energy resolution (1 meV).

## Conclusion

We observed a transient gap generation in BaFe${}_{2}$As${}_{2}$ when the $A_{1g}$ phonon is excited by laser pulses. The transient gap develops at a higher energy than the equilibrium SDW gap and involves a substantial spectral weight redistribution. The feature appears at early-time delays with a short lifetime. The transient gap formation persists up to room temperature, indicating a robust band structure modification in the phonon-driven state, which is likely from an enhanced SDW order as a direct consequence of a transient increasing in the pnictogen height. These observations provide crucial information for future time-resolved investigations on the nature of this newly discovered transient gap. Our finding also opens up new possibilities to study the impact of lattice distortion on superconductivity and nematic order in iron-based superconductors (35, 36) via phonon control of the SDW order.

## Materials and methods

Near-infrared (800 nm) laser pulses with a 40 fs duration were used to excite the $A_{1g}$ phonon in BaFe${}_{2}$As${}_{2}$. The transient optical properties were probed at normal incidence by broadband THz pulses generated by laser-ionized plasma. The THz pulses were detected by EO sampling of the terahertz field in a 100-micron thick GaP and a 300-micron thick GaSe crystal, which cover a detection range from 8 to 28 meV and 34 to 70 meV, respectively. The overall time resolutions are 160 fs for low frequency and 50 fs for high frequency, estimated from the THz pulse width. A long-pass filter was used to remove the scattered pump photons. For each pump-probe time delay, the relative delay between the pump and the EO sampling pulses were kept fixed while scanning the terahertz transient. This ensures that each point in the terahertz probe field detects the material at the same pump-probe time delay. The terahertz probe field and the pump-induced change in the probe field were simultaneously recorded using two lock-in amplifiers. The complex reflection coefficient of the photoexcited sample was calculated using a multilayer model, which models the material as a fully photoexcited top layer of a thickness equal to the near-infrared pump penetration depth (27 nm), and an unexcited bottom layer retaining the equilibrium optical response. The probe penetration depth is frequency dependent and in the order of 200 nm.

## Supplementary Material

pgad164_Supplementary_Data

## Data Availability

All data generated or analyzed during this study are included in this article and its supplementary information files.

## Preprints

This manuscript was posted on a preprint: https://doi.org/10.48550/arXiv.2212.03337.
