# Supplementary material for: Transient gap generation in BaFe2As2 driven by coherent lattice vibrations
Source: PNAS Nexus. 2023 May 16;2(6):pgad164. doi: 10.1093/pnasnexus/pgad164 (PMC10230283; doi:10.1093/pnasnexus/pgad164)
Supplement: pgad164_Supplementary_Data [file pgad164_supplementary_data.pdf]

Supporting Information for

Transient gap generation in $BaFe_2As_2$ driven by coherent lattice vibrations

Jacob A. Warshauer, Daniel Alejandro Bustamante Lopez, Qingxin Dong, Genfu Chen, and Wanzheng Hu

Corresponding Author name.
E-mail: wanzheng@bu.edu

This PDF file includes:

Supporting text
Figs. S1 to S5
Table S1
SI References

Supporting Information Text

Single crystal growth

The $BaFe_2As_2$ single crystals were grown by self-flux method (1). The mixture, with the ratio Ba:FeAs = 1:5, was placed in an alumina crucible with quartz fiber as a cup. The whole assembly was sealed in a silica tube, heated to 1,150 °C for 10 hours, and then slow-cooled to 1,050 °C at the rate of $\sim 2°$ C/hour, after which the silica tube was put into a centrifuge to separate crystals from flux. Typical dimensions of the as-grown single-crystals are $\sim 3mm \times 3mm \times 0.05mm$.

Spectrally resolved measurements of optical response

Multilayer model. The transient complex coefficient of reflection was calculated using a multilayer model. For the optical quantities used here, a single excited layer (SEL) model was utilized; this models the pump and probe interaction with the material as three layers: an air medium n_0, a photo-excited layer with thickness equivalent to the pump penetration depth n_1, and an unexcited layer n_2.

Using equilibrium data, we calculated the complex coefficient of reflection for the unexcited material:

$$r_{\tilde{n}_0,\tilde{n}_2}(\omega) = \frac{\tilde{n}_0(\omega) + \tilde{n}_2(\omega)}{\tilde{n}_0(\omega) - \tilde{n}_2(\omega)}. \quad [1]$$

From this, the bulk-excited transient complex coefficient of reflection is calculated as

$$r^{\text{bulk}}(\omega, t) = \left(1 + \frac{\Delta E}{E}(\omega, t)\right) r_{\tilde{n}_0,\tilde{n}_2}(\omega). \quad [2]$$

In the multilayer model, the effective coefficient of reflection of the configuration of the excited layer and unexcited layer must be equivalent to the bulk approximation:

$$r^{\text{bulk}}(\omega, t) = \frac{r_{\tilde{n}_0,\tilde{n}_1}(\omega, t) + r_{\tilde{n}_1,\tilde{n}_2}(\omega, t)e^{2i\delta(\omega,t)}}{1 + r_{\tilde{n}_0,\tilde{n}_1}(\omega, t)r_{\tilde{n}_1,\tilde{n}_2}(\omega, t)e^{2i\delta(\omega,t)}}, \quad [3]$$

where $\delta(\omega, t) = 2\pi d_p \tilde{n}_1(\omega, t)\omega/c$ and d_p is pump penetration depth. This equation is numerically solved* for the complex index of refraction of the excited layer $\tilde{n}_1$ at each time delay, giving us the transiently excited complex index of refraction $\tilde{n}^{\text{SEL}}(\omega, t)$. From this value, we calculate the transient complex coefficient of reflection $r^{\text{SEL}}(\omega, t)$ and transient optical conductivity $\sigma^{\text{SEL}}(\omega, t)$. Figure S2 shows $\sigma^{\text{SEL}}(\omega, t)$ at $T = 75$ K in both the GaP and GaSe detection regions.

Fitting spectral weight. Transient optical conductivity can be modeled as a meta-stable state at a given time delay. The relaxation dynamics seen in the GaSe detection region of the transient optical conductivity of $BaFe_2As_2$ exhibited three distinct time scales: a long lasting broadening of the Drude taken to be a constant for the probed delays, a fast paced broadening of the Drude T_1, and the very fast gap generation and subsequent destruction T_2. We calculate our change in spectral weight by integrating the real part of the measured transient change in optical conductivity over the energy range of interest:

$$\Delta\mathbf{SW}^{\text{int}}(t) = \int_{\omega_1}^{\omega_2} \Delta\sigma_1(\omega, t)d\omega \quad [4]$$

where $\omega_1 = 34meV$ and $\omega_2 = 43meV$. We fit $\Delta\mathbf{SW}^{\text{int}}$ with the three-term exponential below:

$$\Delta\mathbf{SW}^{\text{fit}}(t) = A\left[1 + \text{Erf}\left(\frac{t}{\Delta t}\right)\right]\left[\sum_{i=0}^{2} C_i e^{-\Gamma_i t}\right] \quad [5]$$

where Δt is the excitation timescale, $A \approx \omega dn(0)e^2/m$, and $\Gamma_i = 1/T_i$ (Γ_0 taken to be 0). The coefficients C_i are used to allocate the spectral weight to each term, so they are subject to the constraint $\sum_{i=0}^{2} |C_i| = 1$. Values in Fig. 3B were produced using fits to each fluence: these fits are seen Fig. S3, and the fitted parameter values are seen in Table S1.

Figure S4 depicts the three-term fit $\Delta\mathbf{SW}^{\text{fit}}$ (green line) to transient change in spectral weight $\Delta\mathbf{SW}^{\text{int}}$ (green diamonds) below T_{SDW}, at 75 K (left), and above T_{SDW}, at 150 K (right). To separate the gap and Drude transient dynamics, $\Delta\mathbf{SW}^{\text{int}}$ is subtracted by the Drude and gap components of the three-term fit:

$$\Delta\mathbf{SW}^{\text{int}}_{\text{gap}}(t) = \Delta\mathbf{SW}^{\text{int}}(t) - A\left[1 + \text{Erf}\left(\frac{t}{\Delta t}\right)\right]\left(C_0 + C_1 e^{-\Gamma_1 t}\right) \quad [6]$$

$$\Delta\mathbf{SW}^{\text{int}}_{\text{Drude}}(t) = \Delta\mathbf{SW}^{\text{int}}(t) - A\left[1 + \text{Erf}\left(\frac{t}{\Delta t}\right)\right]\left(C_2 e^{-\Gamma_2 t}\right). \quad [7]$$

The gap and Drude components of the transient change in spectral weight are plotted in Fig. S4 in blue and red respectively. Carrying a lifetime roughly half that of the fast Drude component, the transient gap coexists with the broadening of the Drude, including when the Drude contribution is maximized. In the inset of Fig. 1C, the transient gap can be seen superimposed on the characteristic broadening of the Drude at $t = 0.75$ ps.

* Inverse Fresnel equation numerically solved using Powell's dog leg method (2)

Oscillatory component. An oscillation was observed in both the high and low frequency detection regions. Measurements with fine time delays were taken at delays following initial excitation. Exponential fits were subtracted from the integrated change in spectral weight to isolate the oscillatory component. The high frequency GaSe detection region behavior can be seen in Fig. S5A. For comparison, Fig. S5B repeats Fig. 4 of the main text.

Table S1. Optimal fit parameters of Equ. 5 on transient change in spectral weight at each fluence. Least squares optimization method used for fitting.

Fluence ($mJcm^{-2}$)	Temp. (K)	T_1 (ps)	T_2 (ps)	A ($10^3\Omega^{-1}cm^{-2}$)	C_0 (a.u.)	C_1 (a.u.)	C_2 (a.u.)
0.53	75	0.44 $\pm$0.10	0.19 $\pm$0.01	18.83 $\pm$0.52	0.041 $\pm$0.001	0.480 $\pm$0.014	-0.479 $\pm$0.014
1.12	75	0.46 $\pm$0.17	0.20 $\pm$0.03	18.34 $\pm$0.92	0.085 $\pm$0.004	0.500 $\pm$0.025	-0.415 $\pm$0.021
2.13	75	0.42 $\pm$0.16	0.22 $\pm$0.04	30.53 $\pm$2.38	0.071 $\pm$0.006	0.494 $\pm$0.039	-0.435 $\pm$0.034
3.35	75	0.40 $\pm$0.19	0.22 $\pm$0.04	42.13 $\pm$3.98	0.059 $\pm$0.005	0.465 $\pm$0.044	-0.476 $\pm$0.045
0.53	150	0.39 $\pm$0.21	0.25 $\pm$0.02	10.20 $\pm$0.27	0.038 $\pm$0.001	0.557 $\pm$0.015	-0.405 $\pm$0.011

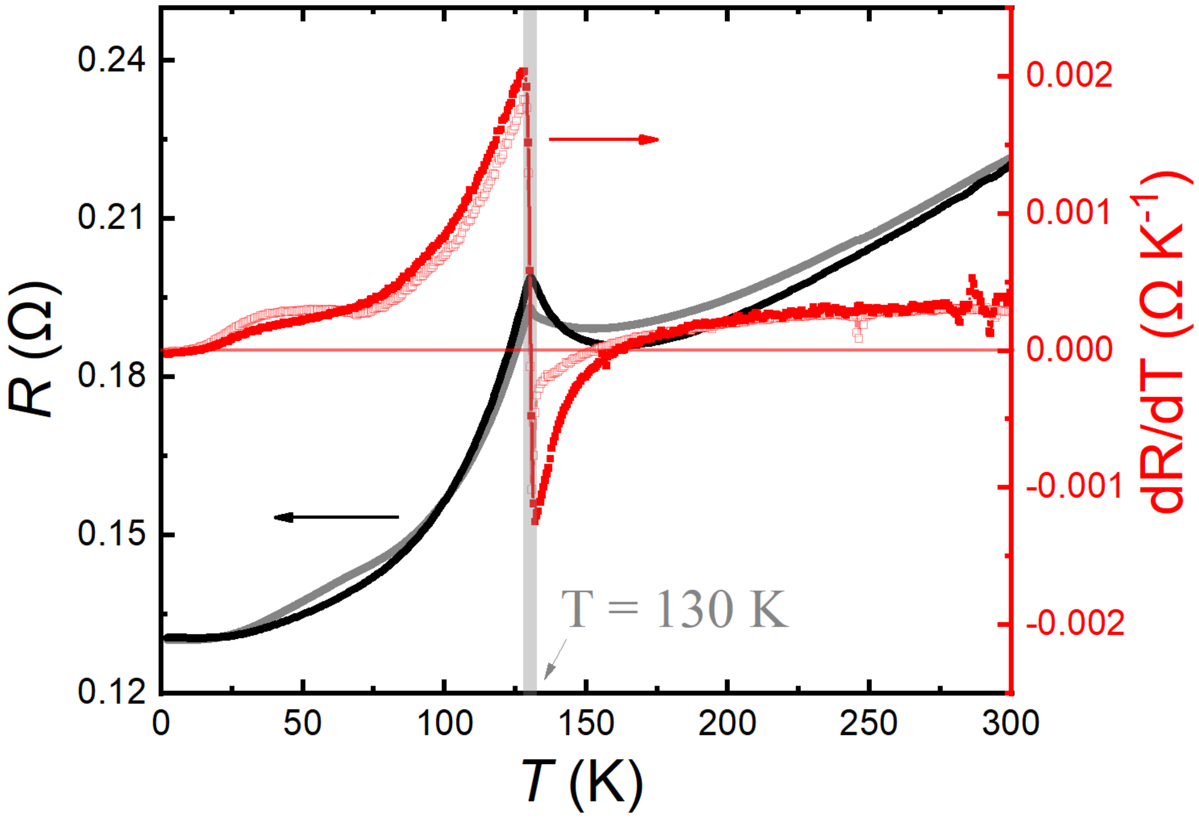

Fig. S1. The temperature dependence of resistance (solid lines) and the first-order derivative (squares) for $BaFe_2As_2$ obtained on two samples from the same batch. A clear change caused by SDW can be seen at $T \sim 130$ K.

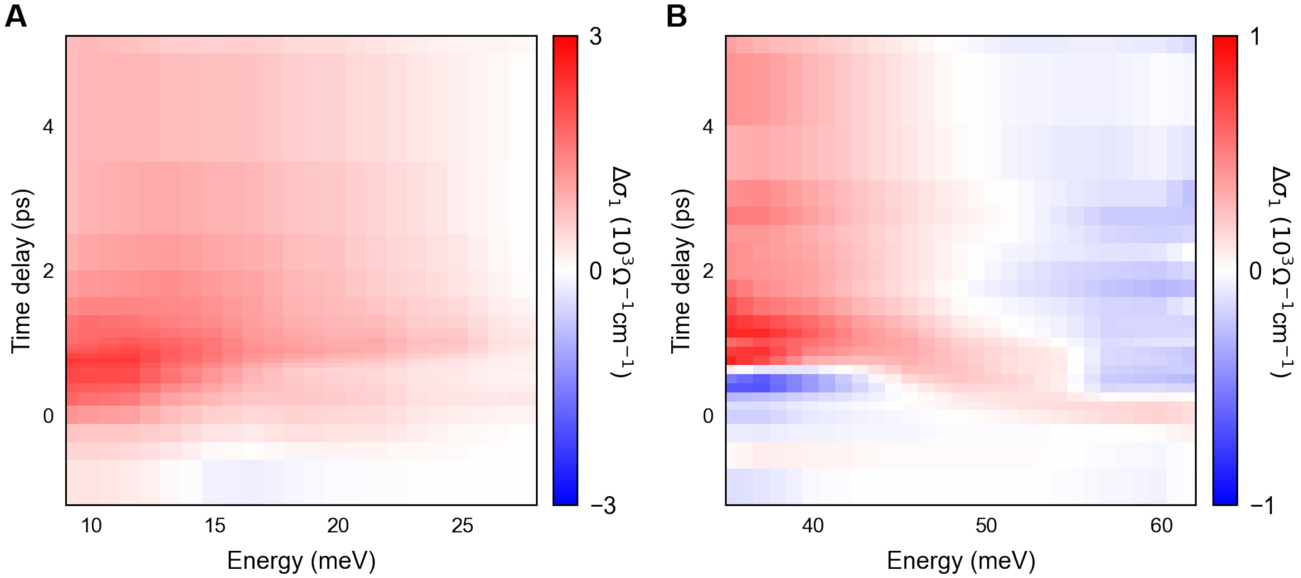

Fig. S2. Light-induced change in optical conductivity $\Delta\sigma_1(\omega, t)$ over GaP **A** and GaSe **B** detection regions. Measurements conducted at T = K with pump fluence 0.53 mJ/cm^2.

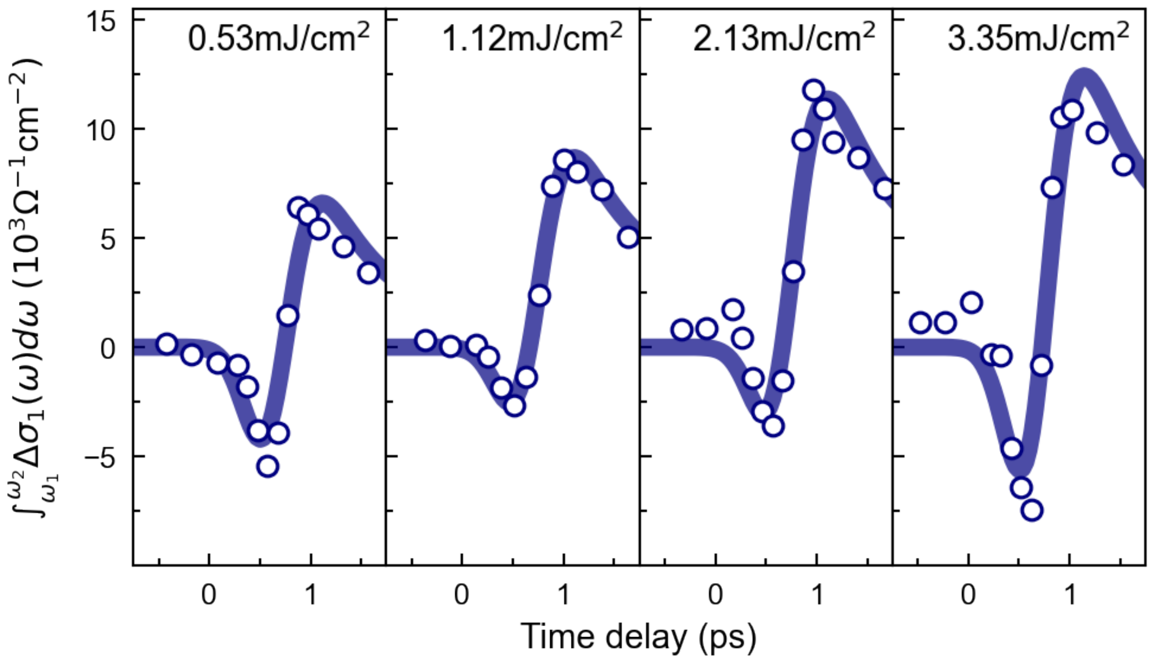

Fig. S3. The fits (solid lines), using Equ. 5, to the raw transient change in spectral weight (circles), Equ. 4. Measurements conducted at T = 75 K. The lower and upper bounds of integration are ω_1 = 34 meV and ω_2 = 43 meV.

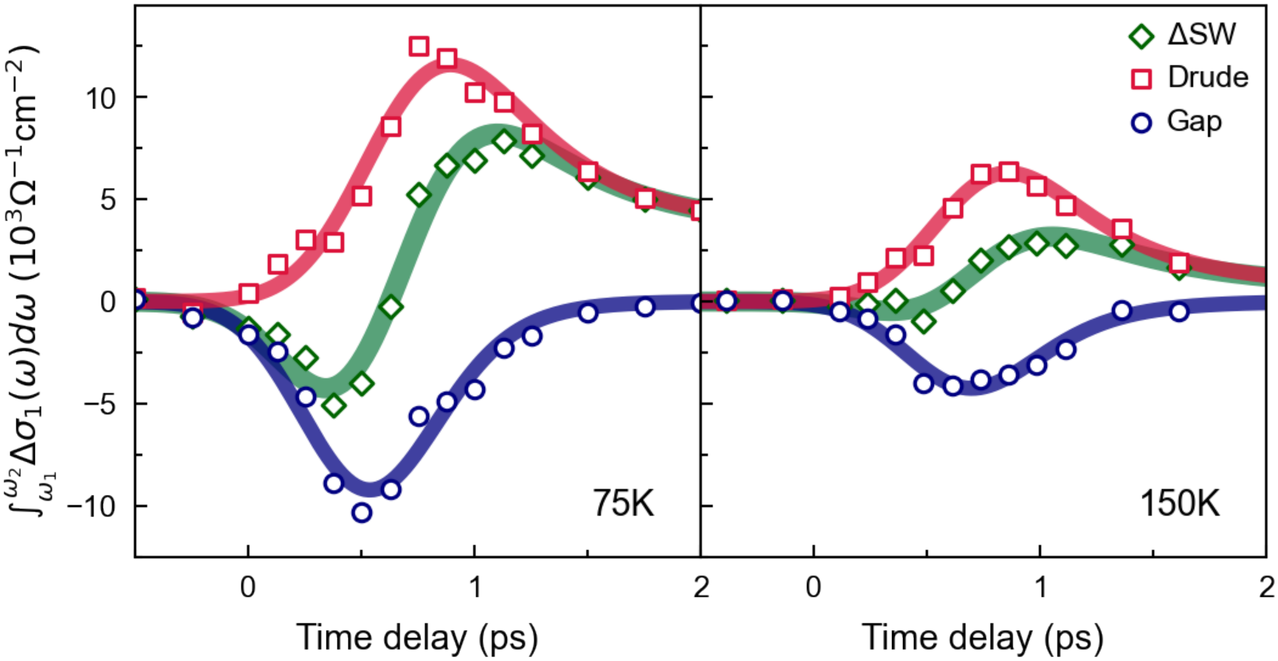

Fig. S4. The transient change in the spectral weight (green diamonds), Drude component (red squares), and the transient gap component (blue cirles) at T = 75 K (left) and T = 150 K (right). Integration was done over ω_1 = 34 meV to ω_2 = 43 meV Solid lines are the three-term fit to the transient change in spectral weight (green line); two-term Drude component (red line), corresponding to the right-most term in Equ. 7; and single-term transient gap component (blue line), corresponding to the right-most term in Equ. 6.

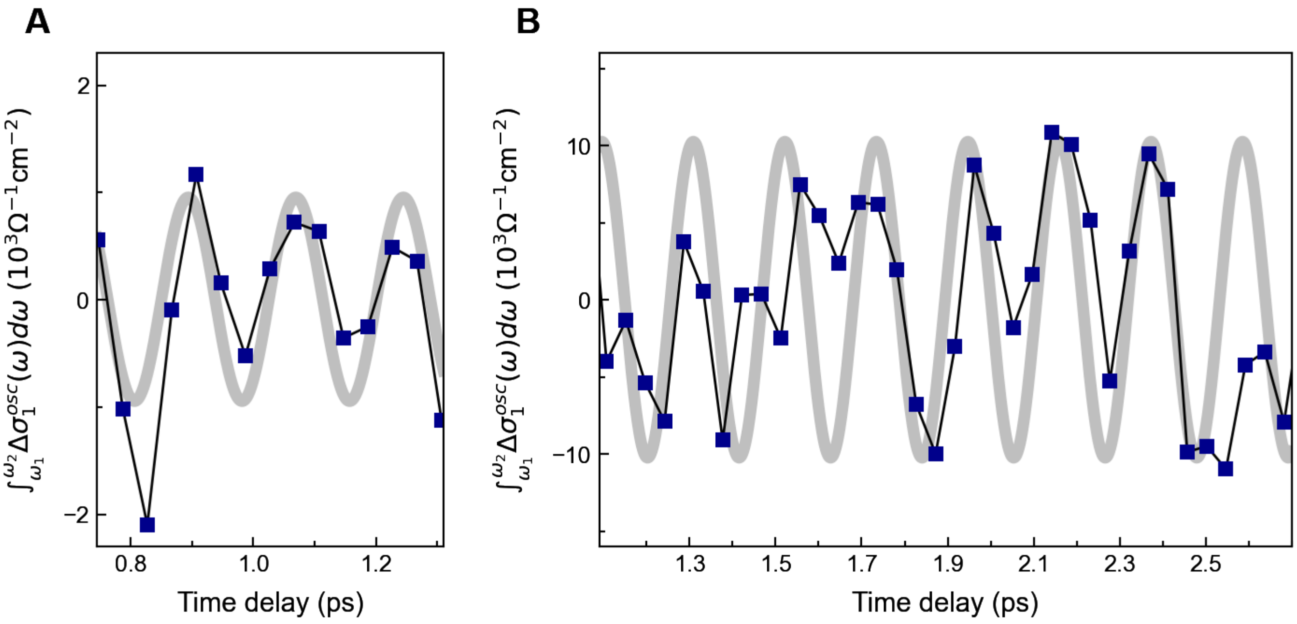

Fig. S5. Oscillatory behavior seen in high and low frequency detection regions. The navy squares are the integrated $\int_{\omega_1}^{\omega_2} \Delta\sigma_1(\omega)d\omega$, with the displacive component subtracted. **A**, High frequency GaSe detection region measurements taken at 134 K with limits of integration set at ω_1 = 45 meV and ω_2 = 51 meV. A sinusoidal function with 5.7 THz oscillation frequency is seen in grey. **B**, Low frequency GaP detection region measurements taken at 120 K with limits of integration set at ω_1 = 12 meV and ω_2 = 20 meV. A sinusoidal function with 5 THz oscillation frequency is seen in grey.

References

1. Y Nakajima, T Taen, T Tamegai, Possible superconductivity above 25 K in single-crystalline Co-doped $BaFe_2As_2$. *J. Phys. Soc. Jpn.* **78**, 023702–023702 (2009).
2. MJD Powell., A hybrid method for nonlinear equations. *In: Robinowitz, P., (Ed.), Numer. Methods for Nonlinear Algebr. Equations, Gordon Breach Sci. Lond.* pp. 97–144 (1970).
